# Supplementary material for: Subcycle surface electron emission driven by strong-field terahertz waveforms
Source: Nat Commun. 2023 Oct 18;14:6596. doi: 10.1038/s41467-023-42316-0 (PMC10584819; doi:10.1038/s41467-023-42316-0)
Supplement: Supplementary file 1 — Supplementary Information [file 41467_2023_42316_MOESM1_ESM.pdf]

Subcycle surface electron emission driven by strong-field terahertz waveforms: supplementary information

Shaoxian Li[1,2], Ashutosh Sharma[3], Zsuzsanna Márton[3,4], Priyo S. Nugraha[1,5], Csaba Lombosi[1], Zoltán Ollmann[1,4], István Márton[6,7], Péter Dombi[3,6], János Hebling[1,4,5], József A. Fülöp[1,3]

[1]Szentágothai Research Centre, University of Pécs, 7624 Pécs, Hungary
[2]Center for Terahertz Waves and College of Precision Instrument and Optoelectronics Engineering, and the Key Laboratory of Optoelectronics Information and Technology, Ministry of Education, Tianjin University, Tianjin 300072, China
[3]ELI-ALPS Research Institute, ELI-HU Non-Profit Ltd., 6728 Szeged, Hungary
[4]Institute of Physics, University of Pécs, 7624 Pécs, Hungary.
[5]HUN-REN-PTE High-Field Terahertz Research Group, 7624 Pécs, Hungary
[6]Wigner Research Centre for Physics, 1121 Budapest, Hungary
[7]Institute for Nuclear Research (Atomki), 4001 Debrecen, Hungary
Correspondence and requests for materials should be addressed to J.A.F. (email: jozsef.fulop@eli-alps.hu)

Calibration of the THz field polarity

The THz field polarity was calibrated by comparison with a static (DC) field. A Y-cut $LiNbO_3$ (LN) crystal with $L = 1\ \mathrm{mm}$ thickness was used as the electro-optic material. Using the insulator LN, rather than the semiconductor GaP, enabled to apply a large DC voltage across the crystal. To facilitate such comparison, the relation between the (DC or THz) electric field and the field-induced phase retardation (i.e. the difference in phase shift between orthogonally polarized components) of the optical sampling pulse has to be established. The field-induced phase retardation is given by

$$\phi = \frac{\omega}{c}\int_0^L \Delta n_E(z)dz = \frac{\omega}{c}R\int_0^L E(z)dz, \qquad (1)$$

where $\Delta n_E(z) = R \cdot E(z)$ is the electric-field-induced change of the optical refractive index at position z within the LN crystal, ω is the mean frequency of the sampling pulse, and c is the velocity of light in vacuum. Implicit in this relation is the assumption that the optical sampling pulse is sufficiently short such that $E(z)$ is well defined even in case of a varying field. This condition is fulfilled here to a good approximation, because the 200 fs optical pulse duration is much shorter than the oscillation cycle of the THz field (about 2.6 ps). R is a constant, which depends on the type of the electro-optic crystal, the propagation and polarization directions of the optical sampling pulse, and the polarization of the THz or DC field. Furthermore, it is assumed that the THz pulse propagates into the same direction as the optical pulse, which is the Y-direction in LN in the present case. Under conventional balanced-detection conditions, as was the case here, the detected EOS signal is proportional to the phase change given by Supplementary Equation (1).

In the present case, the DC and THz fields were polarised along the optic axis (Z-direction) of the LN crystal. The polarization of the optical pulse was along the $45°$ polar angle direction, measured from the Z-axis in the XZ-plane. For this situation

$$R = \frac{1}{2}(n_o^3 r_{13} - n_e^3 r_{33}) \tag{2}$$

holds [1, 2], where n_o is the ordinary refractive index of LN at the optical wavelength and r_{13} denotes the free and the clamped electro-optic coefficients in the case of the DC and the THz fields, respectively [3, 4].

Static (DC) field. Because it is only the relative direction of the static and THz fields which is of interest here, it was assumed in the calculation that the static field imposed by the HV supply is homogeneous over the length of the LN crystal and has the field strength of E_{DC}. Substitution of $E(z) \equiv E_{DC}$ into Supplementary Equation (1) and rearranging gives

$$E_{DC} = \frac{1}{\omega R}\frac{c}{L}\phi. \tag{3}$$

THz field. In this case, a similar calculation can be carried out as outlined in Refs. [1, 2]. The important difference is that the relative sign of the THz field (more precisely, its derivative; see below) and the corresponding phase retardation has to be traced accurately. The detailed calculation yields for the time derivative of the phase retardation the following relation:

$$\frac{d\phi}{dT} = -\frac{\omega R}{\Delta n}E(T) + \frac{\omega R}{\Delta n}E\left(T + \frac{\Delta n L}{c}\right)e^{-\alpha L} + \frac{\alpha c}{\Delta n}\phi(T). \tag{4}$$

The time T is measured by the delay of the optical pulse with respect to the THz pulse. The difference between the optical group index and the THz refractive index in LN is $\Delta n = n_g(\omega) - n_{THz} \approx 2.3 - 4.9 = -2.6$. Obviously, the probe pulse propagates much faster through the crystal than the THz pulse and sweeps over the portion of the THz pulse that lies inside the crystal. The first and second terms at the right-hand side of Supplementary Equation (4) are of opposite signs and they originate from the input and exit regions of the LN crystal, respectively. In the experiment, these two contributions were clearly separated in time by about 9 ps and only the first term was considered for the field polarity calibration. The third term in Supplementary Equation (4) could be ignored because of the small absorption of LN at the low THz frequencies encountered in the experiment [1]. Thus, to a good approximation, the THz field was related to the derivative of the measured phase retardation as follows:

$$E(T) \approx -\Delta n \frac{1}{\omega R}\frac{d\phi}{dT}. \tag{5}$$

By comparing Supplementary Equations (3) and (5), and taking into account that $\Delta n < 0$, the vector directions (polarity) of the DC field and the THz field at a time instant T are identical if the phase retardation measured with the DC field and the time derivative of the phase retardation measured with THz field have the same signs. The field polarities are opposite if the signs are opposite. Here, it is important to recall that T is defined as the delay of the optical sampling pulse with respect to the THz pulse. Hence, T is positive when the sampling pulse lags behind the THz pulse, and negative when it is preceding the THz pulse.

Characterization of the BeO surface

The BeO cathode surface was characterized by optical microscopy and by atomic force microscopy (AFM). The surface topography data from AFM were used as input for numerical calculations of the THz near field, as well as for calculating the distributions of the polar and azimuthal angles of the local surface normal vectors and the roughness parameters. Furthermore, the vertical structure of the surface was investigated by focused ion beam milling and subsequent scanning electron microscopy (SEM). As mentioned in the “Methods” section of the main article, the cathode in the experiment was

the first dynode of a commercial electron multiplier (Hamamatsu R595, see Ref. [5]). The material of the dynode was specified by the manufacturer as Cu-BeO, where BeO is coated onto a Cu substrate electrode [6].

Optical microscopy. The surface of the cathode was inspected by optical microscopy at different magnifications using an Olympus DSX510 digital optical microscope. The microscope images show a surface with significant roughness on different scales (Supplementary Figure 1). The size of the resolved surface structures ranges from the 100-µm scale to the µm scale. 3D imaging can increase the depth of focus and thus the image quality in 2D, but the resolution and reliability of height measurement were not sufficient to characterise the nanometre-scale surface roughness quantitatively.

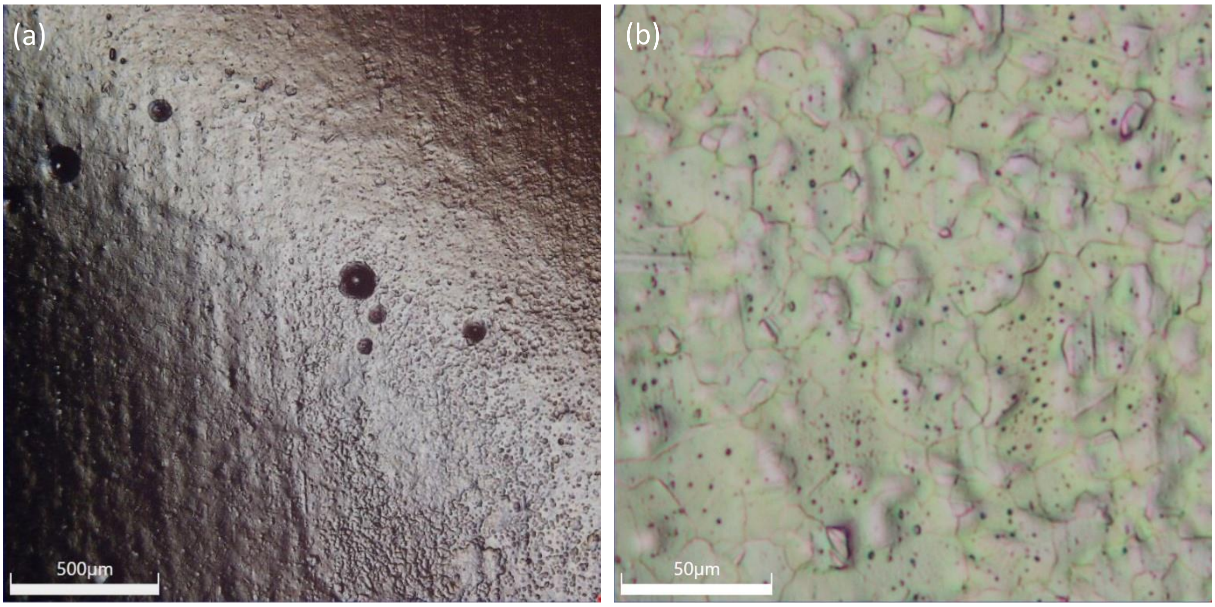

Supplementary Figure 1. Optical microscope images of the BeO cathode surface with magnifications of 139× (a) and 1387× (b).

Scanning electron microscopy (SEM). The depth structure of the cathode surface was investigated by milling a trench into it using the focused ion beam technique (Supplementary Figure 2a), and subsequently imaging by SEM. This inspection showed that the BeO coating layer has a thickness of about 140 nm (Supplementary Figure 2b), with local variations within the range of about 100 nm to 200 nm.

Atomic force microscopy (AFM). In order to quantify the surface roughness on the µm-nm length scales, relevant for calculating the THz near field, topography measurements were carried out using AFM. At different positions on the surface, 30 µm × 30 µm areas were measured with a resolution of 150 nm. One example for the topography measurement is shown in Figure 3a in the main article. Characteristic features are grains with typical lateral sizes of 1 µm to 2 µm and about 50 nm to 150 nm maximum elevations above the surrounding surface. According to the numerical calculations of the THz near field (see the main article), the field enhancement is highest at such structures.

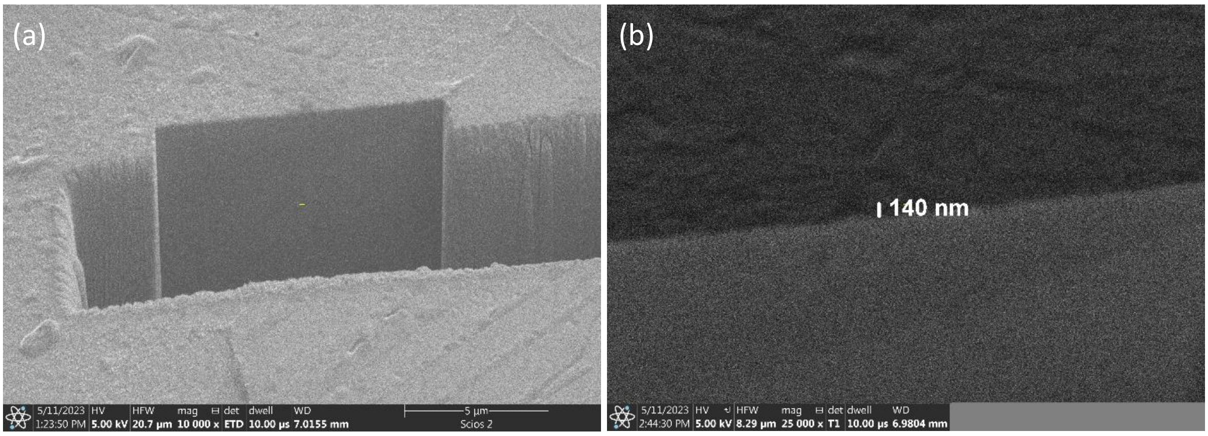

Supplementary Figure 2. (a) SEM image of a trench in the cathode surface, produced by focused ion beam milling. A magnification of 10 000× was used. (b) An SEM image of the trench edge with a magnification of 25 000× shows the BeO coating layer (darker upper part) and the substrate (lighter bottom part).

Surface roughness and anisotropy. Measuring the surface topography by AFM enabled the estimation of the angular deviation of the local surface normals from the mean surface normal. Local surface normals were calculated from the surface topography data. The distribution of the spherical polar angles α, defined by the normals of the local surface elements relative to the normal of the mean surface plane, is plotted in Supplementary Figure 3a for the surface part shown in Figure 3a of the main article. The mean value of the local polar angles is $\langle\alpha\rangle = 2.0°$ and the angle bracket means surface average. The polar angle distribution is dominated by small (≲5°) angles, but large (10° to 20°) angles also occur.

The surface roughness parameter $\bar{\alpha}$ is defined by the relation [7]

$$\sin^2 \bar{\alpha} = \int_0^{\pi} f(\alpha) \sin^2 \alpha \, d\alpha, \tag{6}$$

where $f(\alpha)$ is the distribution of the spherical polar angles α, normalized according to $\int_0^{\pi} f(\alpha) d\alpha = 1$. For the entire surface part shown in Figure 3a of the main article, $\bar{\alpha} = 0.3°$. It is important to note that the electron emission was dominated by emission hot spots, where the field enhancement was largest. Therefore, the effective emitting surface is a small fraction of the total surface and concentrates around grains (see Figure 3 in the main article). At such locations, larger polar angles are more frequent (Supplementary Figure 3c). Consequently, the effective value of the surface roughness parameter (averaged over the effective emitting surface) for electron emission was significantly larger than the roughness averaged over the entire surface. For the example shown in Figure 3 of the main article, the effective value of the surface roughness parameter was $\bar{\alpha}_{\text{eff}} = 1.4°$. We note that emission hot spots due to local field enhancement were described recently in edge emitters [8]. Supplementary Figure 3b shows the distribution of the azimuthal angles of the local surface normal. Based on this distribution, some surface anisotropy may be present which can also affect the electron emission characteristics.

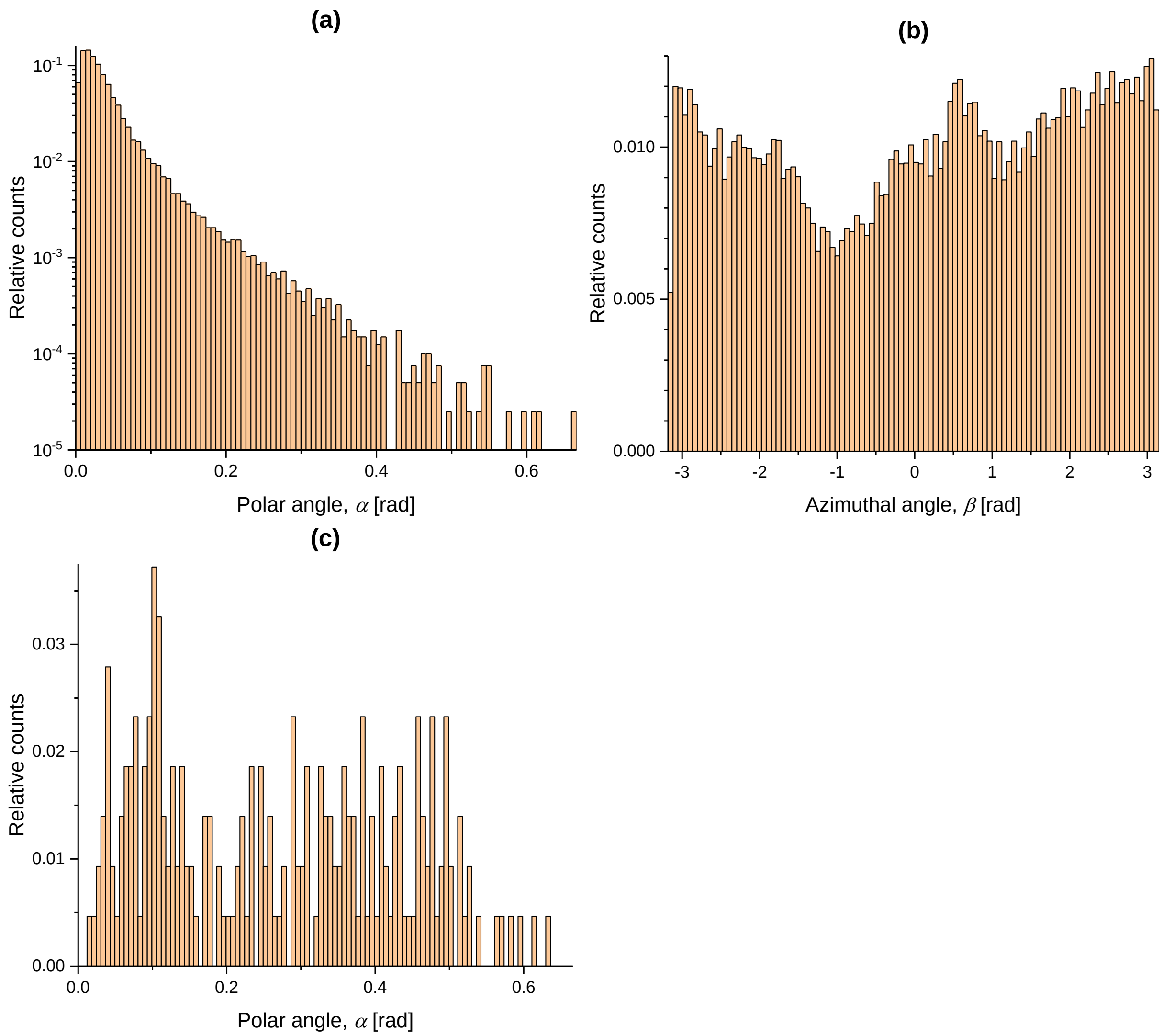

Supplementary Figure 3. Distribution of the α polar (a) and the β azimuthal (b) angles of the local surface normal for the surface shown in Figure 3a in the main article, determined from the AFM measurement. (c) Polar angle distribution for the emitting hot spots.

Dependence of electron emission on the THz-field polarization

The polarization angle dependence of the surface-normal electric field component is influenced by the surface roughness (see section "Theoretical model of the field-driven electron emission" below) [7]. Because the electron emission is determined by the surface-normal field component, it can be expected that measuring the polarization dependence of the emission signal enables the determination of the roughness parameter $\bar{\alpha}$ (see section "Characterization of the BeO surface" above) and gives a more complete characterization of how the cathode surface conditions determine the electron emission process. Therefore, the measurement of the polarization-angle dependence of the emission signal was carried out. We note that in the past, the polarization dependence of the photoemission from a metal surface was studied with picosecond laser pulses and it was used to identify surface multiphoton photoeffect as the emission mechanism, in contrast to volume photoeffect or thermionic emission [9].

The observed electron emission signal S, as function of the polarization angle φ, is shown in Supplementary Figure 4a for the peak values of the THz electric field strength of $E_0 = 68$ kV/cm, 74 kV/cm, 84 kV/cm, and 100 kV/cm, corresponding to values of the Keldysh $\gamma \approx 2.4$, 2.2, 1.9, and

1.6, respectively. The data in the range $\varphi = -180°$ to $\varphi = -120°$ are missing because of geometric constraints on the chamber rotation due to the vacuum connection. The most notable feature is the double maximum of the emission current, which was observed at all field strengths. The two maxima are observed at about $\varphi = -18°$ and $\varphi = 71°$. A local minimum between the two maxima is located at about $\varphi = 35°$.

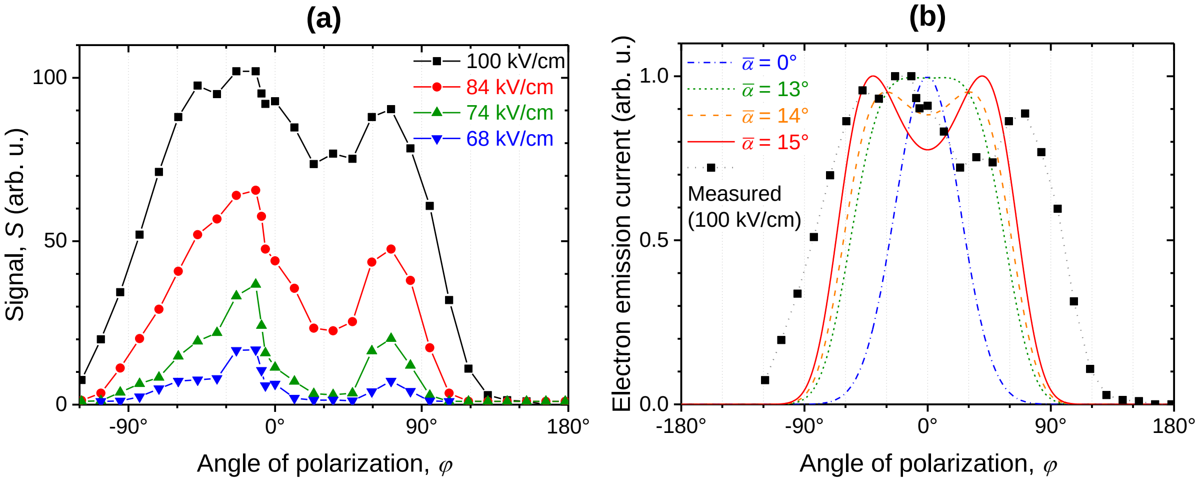

Supplementary Figure 4. Polarization angle dependence of electron emission. (a) Measured electron emission signals as functions of the polarization angle, recorded at four different THz peak electric field strengths E_0. (b) Calculated electron emission current as function of the polarization angle for different values of the surface roughness parameter $\bar{\alpha}$. For comparison, the symbols show the measured data for $E_0 = 100$ kV/cm electric field. Both the calculated and the measured data are normalized to a maximum of 1 for better comparison.

For a qualitative interpretation of the experimental results, a theoretical model was used, which takes into account the effect of surface roughness (see section "Theoretical model of the field-driven electron emission" below). In the calculation, the measured THz waveform was used as the (quasistatic) driving field in the Fowler-Nordheim model and the work function of the BeO cathode surface was 4.1 eV. Supplementary Figure 4b shows the calculated emission current as function of the polarization angle for different values of the surface roughness parameter $\bar{\alpha}$ and in comparison to the measured data for $E_0 = 100$ kV/cm THz peak electric field. For smoother surfaces with roughness parameter values of about 10° or smaller, a single current peak is predicted. For larger roughness parameter values of about $\bar{\alpha} \approx 15°$, the calculation shows double maxima in the emission current, appearing around $\varphi \approx \pm 40°$. Because the effective roughness parameter $\bar{\alpha}_{\text{eff}} = 1.4°$, obtained from the analysis of the AFM data (see section "Characterization of the BeO surface"), is about ten times smaller, it is not sufficient to explain the observed double maxima. As a conclusion of this investigations, it was found that the polarization angle dependence of the electron emission signal is not well suited to reliably quantify the surface roughness. Surface anisotropy (see section "Characterization of the BeO surface" above) may contribute to the $S(\varphi)$ dependence in Supplementary Figure 4a. However, the investigation of such effects is beyond the scope of this study.

Theoretical model of the field-driven electron emission

Fowler-Nordheim model. THz-field-driven electron emission from the cathode surfaces was described by the Fowler-Nordheim model [10] to estimate the yield of the emitted electrons. In the quasi-static approximation, the instantaneous tunnelling current density J can be given by the Fowler-Nordheim equation [10-12]

$$J = \frac{aE^2}{W\tau^2} \cdot \exp\left(-vb\frac{W^{3/2}}{E}\right). \quad (7)$$

Here, E is the surface-normal component of the total THz electric field (the sum of the incoming and the reflected fields), whereby only the active half-cycle of the THz pulse is considered. Supplementary Equation (7) gives the emission current in the negative half-period of the field, i.e. when it points into the cathode material. There is no electron emission in the positive half-cycle. As before, W is the work function of the cathode material. The Fowler-Nordheim constants a and b are defined as $a = e^3/(16\pi^2\hbar)$ and $b = (4/3)\sqrt{2m_e}/(e\hbar)$, respectively. Here, e is the elementary charge, m_e is the electron mass, and $\hbar$ is the reduced Planck constant. The quantities $v = v(f)$ and $\tau = \tau(f)$ are functions of the normalized field strength $f = E/E_W$, where $E_W = 4\pi\varepsilon_0 W^2/e$ is the critical field which lowers the potential barrier to zero relative to the Fermi energy. In the low-field limit of $E \ll E_W$ (i.e. when $f \ll 1$), $v(f) \approx 1 - f + (f/6)\ln(f)$ and $\tau(f) \approx 1 + f/9 - (f/18)\ln(f)$ hold to a good approximation. In the present calculations, the approximate values $v = \tau = 1$ were used.

Electron backscattering. The solution of the classical equation of motion in the single-cycle THz field indicated that the electrons emitted from the cathode during the leading half cycle (corresponding to $\varphi = 180°$ polarization angle) follow returning trajectories. Upon returning to the cathode, the electrons either recombine within the cathode or they are backscattered with a probability given by the electron backscattering coefficient. The analysis of the THz-driven electron emission data (Supplementary Figure 5 and Figure 2b in the main article) in the present work lead to an estimation of $\sigma = 9\%$ for the backscattering coefficient of the BeO cathode (see "Discussion" section in the main article).

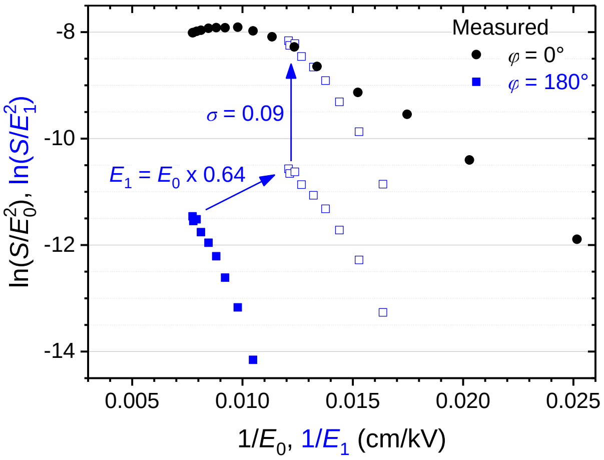

Supplementary Figure 5. Fowler-Nordheim plot of the measured data (full symbols, same data as in Figure 2a in the main article). The blue arrows and the empty blue squares show the measured data for $\varphi = 180°$ with the electric field strength scaled only by the half-cycle asymmetry factor of 0.64, and with an electron backscattering coefficient of $\sigma = 0.09$ in addition to the same half-cycle asymmetry.

In order to give an independent estimation of the electron backscattering effect, the time-dependent Schrödinger equation, in one spatial dimension, was numerically integrated. The time evolution of the square of the absolute value of the wave function, for $\varphi = 180°$ THz field polarity and $E_0 = 100$ kV/cm amplitude, shows the signature of backscattering (Supplementary Figure 6). The contribution of backscattering was estimated by comparing the emitted electron current for a sine-like pulse with equal field maxima in the leading and trailing half cycles. Backscattering probabilities of about 15% were obtained. This value compares well with the estimation from the experiment. Whereas literature data on electron backscattering coefficient of BeO are sparse, coefficient values of 1% to 10% were given for beryllium, based on Monte Carlo simulations for energies above 100 eV [13].

For tungsten, based on a partial-wave analysis for scattering at a muffin-tin potential, $\lesssim 20\%$ coefficient value was given [14]. These values compare well with the values obtained here.

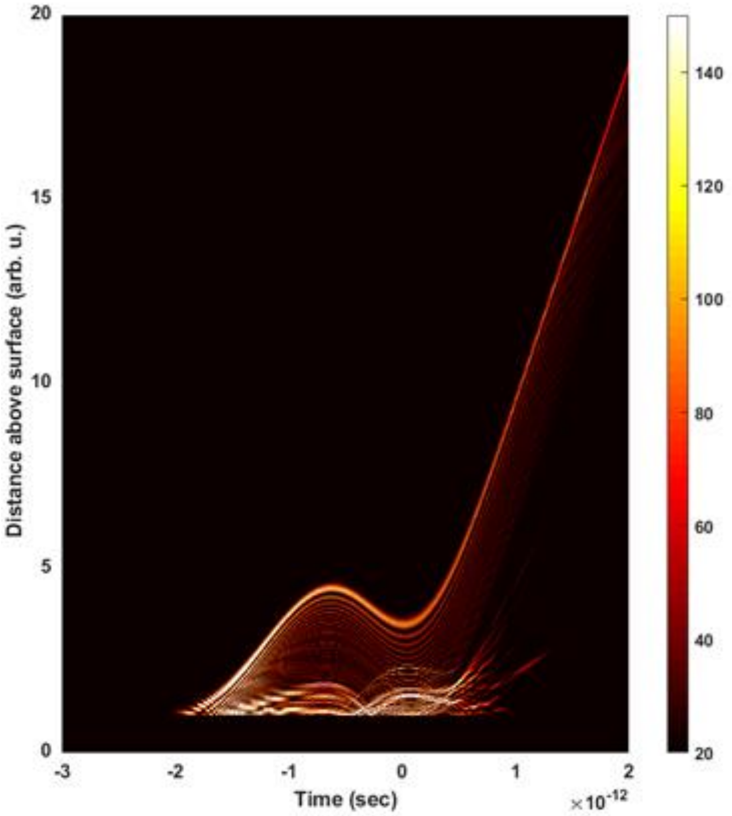

Supplementary Figure 6. Time evolution for the square of the absolute value of the wave function obtained by the numerical solution of the time-dependent Schrödinger equation in one spatial dimension. The THz field polarity was $\varphi = 180°$ and the field amplitude 100 kV/cm.

Surface roughness. In order to investigate the polarization angle dependence of THz-induced emission current, the theoretical model from Grant and Cutler [7] was used. They analysed the influence of surface roughness on the electron emission yield and introduced the surface roughness parameter $\bar{\alpha}$ as defined above (see the section "Characterization of the BeO surface"). For a perfectly smooth plane surface ($\bar{\alpha} = 0$), the effective field component is the one perpendicular to the surface, $E_{\perp} = E_{\mathrm{p}} \sin(\theta)$. Here, E_{p} is the p-polarized field component and θ is the angle of incidence of the THz beam. The electric field components and the angles are defined in Figure 1b of the main article. For a surface with finite roughness ($\bar{\alpha} > 0$), the surface normal $\mathbf{n}$ becomes a position-dependent local quantity $\mathbf{n} = \mathbf{n}(x, y)$ (see also section "Characterization of the BeO surface" above). Consequently, the average effective component of the field is modified, so that the s-polarized component E_{s} can also contribute (it can have a nonzero average component along the local surface normal). The average value of $(\mathbf{n} \cdot \mathbf{E})^2$ can be calculated as follows:

$$\langle(\mathbf{n} \cdot \mathbf{E})^2\rangle = \frac{1}{2} {E_{\mathrm{s}}}^2 \sin^2\bar{\alpha} + {E_{\mathrm{p}}}^2 \left(\cos^2\bar{\alpha}\,\sin^2\theta + \frac{1}{2}\sin^2\bar{\alpha}\,\cos^2\theta\right), \qquad (8)$$

where $\mathbf{E}$ is the total THz electric field and θ is measured from the mean surface normal $\langle\mathbf{n}\rangle$. For a perfectly smooth surface, $\bar{\alpha} = 0$ and Supplementary Equation (8) reduces to $E_{\mathrm{p}}^2 \sin^2\theta$. Note that E_{s} and E_{p} are dependent on the polarization angle φ, and so is $\langle(\mathbf{n} \cdot \mathbf{E})^2\rangle$. Supplementary Equations (7) and (8) can be used to estimate the electron yield as function of φ.

The surface roughness affects the intensity of electron emission in two ways. Firstly, the surface roughness modifies the average surface-normal component of the electric field, as indicated by Supplementary Equation (8). For example, for a p-polarized THz pulse (corresponding to $\varphi = 0°$ or $\varphi = 180°$ polarization angles), the average normal field component is reduced, as compared to a perfectly smooth surface. This causes a reduction of the electron emission current. However, this effect is negligibly small for the roughness parameters of the cathode used here. Secondly, the surface roughness causes an effective field enhancement of about 2 for a p-polarized THz pulse in the present case, which results in a large, several orders of magnitude increase of the emission current. This second effect dominates over the first one for the p-polarized field component, resulting in a net current increase as compared to a smooth surface.

References

[1] P. U. Jepsen, C. Winnewisser, M. Schall, V. Schyja, S. R. Keiding, H. Helm, Detection of THz pulses by phase retardation in lithium tantalate, *Physical Review E* **53**, R3052–R3054 (1996).

[2] C. Winnewisser, P. U. Jepsen, M. Schall, V. Schyja, H. Helm, Electro-optic detection of THz radiation in $LiTaO_3$, $LiNbO_3$ and ZnTe, *Applied Physics Letters* **70**, 3069–3071 (1997).

[3] B. E. A. Saleh, M. C. Teich, *Fundamentals of photonics* (Wiley Interscience, Hoboken, N. J., 2007).

[4] G. G. Gurzadian, V. G. Dmitriev, D. N. Nikogosian, *Handbook of nonlinear optical crystals* (Springer, New York, 1997).

[5] *Electron multipliers*. Available online: https://www.hamamatsu.com/content/dam/hamamatsu-photonics/sites/documents/99_SALES_LIBRARY/etd/EMT_TPMH1354E.pdf (accessed on 10 May 2023).

[6] *Photomultiplier tubes. Basics and applications*. Available online: https://www.hamamatsu.com/content/dam/hamamatsu-photonics/sites/documents/99_SALES_LIBRARY/etd/PMT_handbook_v4E.pdf (accessed on 10 May 2023).

[7] D. Grant, P. H. Cutler, Surface roughness and photoemission, *Physical Review Letters* **31**, 1171–1174 (1973).

[8] T. Paschen, R. Roussel, L. Seiffert, B. Kruse, C. Heide, P. Dienstbier, J. Mann, J. Rosenzweig, T. Fennel, P. Hommelhoff, Ultrafast strong-field electron emission and collective effects at a one-dimensional nanostructure, *ACS Photonics* **10**, 447–455 (2023).

[9] L. A. Lompre, J. Thebault, G. Farkas, Intensity and polarization effects of a single 30-psec laser pulse on five-photon surface photoeffect of gold, *Applied Physics Letters* **27**, 110–112 (1975).

[10] R. H. Fowler, L. Nordheim, Electron emission in intense electric fields, *Proceedings of the Royal Society of London. Series A, Containing Papers of a Mathematical and Physical Character* **119**, 173–181 (1928).

[11] E. L. Murphy, R. H. Good, Thermionic emission, field emission, and the transition region, *Physical Review* **102**, 1464–1473 (1956).

[12] R. G. Forbes, Simple good approximations for the special elliptic functions in standard Fowler-Nordheim tunneling theory for a Schottky-Nordheim barrier, *Applied Physics Letters* **89**, 113122 (2006).

[13] A. Hussain, L. Yang, S. Mao, B. Da, K. Tőkési, Z. J. Ding, Determination of electron backscattering coefficient of beryllium by a high-precision Monte Carlo simulation, *Nuclear Materials and Energy* **26**, 100862 (2021).

[14] G. Wachter, C. Lemell, J. Burgdörfer, M. Schenk, M. Krüger, P. Hommelhoff, Electron rescattering at metal nanotips induced by ultrashort laser pulses, *Physical Review B* **86**, 035402 (2012).
